# Resilient Together Dyadic Intervention for Persons With Dementia and Their Partners: Protocol for a Feasibility Randomized Controlled Trial

**DOI:** 10.2196/106005

**Published:** 2026-10-27

**Authors:** Sarah M Bannon, Sydney M McCage, Renata Elizalde Carrillo, Stephan A Brandt, Grace O Allison, Aida Aminpour, Kristin Walker, Talea Cornelius, Robert A Parker, Kristen Dams-O'Connor, Bradford Dickerson, Christine Ritchie, Ana-Maria Vranceanu

**Affiliations:** 1 Department of Rehabilitation Medicine and Human Performance, Brain Injury Research Center, Icahn School of Medicine at Mount Sinai, 5 East 98th Street, New York, NY, 10029, United States, 1 2122415152; 2 Department of Neurology, Icahn School of Medicine at Mount Sinai, New York, NY, United States; 3 Department of Geriatrics and Palliative Medicine, Icahn School of Medicine at Mount Sinai, New York, NY, United States; 4 Department of Psychology, Stony Brook University, Stony Brook, NY, United States; 5 Department of Psychology, McGill University, Montreal, QC, Canada; 6 Department of Medicine, Columbia University Irving Medical Center, New York, NY, United States; 7 Department of Medicine Harvard Medical School, Biostatistics Center, Massachusetts General Hospital, Boston, MA, United States; 8 Departments of Neurology and Psychiatry, Frontotemporal Disorders Unit, Massachusetts General Hospital, Boston, MA, United States; 9 Department of Medicine Harvard Medical School, Mongan Institute Center for Aging and Serious Illness and the Division of Palliative Care and Geriatric Medicine, Massachusetts General Hospital, Boston, MA, United States; 10 Department of Psychiatry, Center for Health Outcomes and Interdisciplinary Research, Massachusetts General Hospital, Boston, MA, United States

**Keywords:** dementia, dyad, emotional distress, intervention, diagnosis, telehealth

## Abstract

**Background:**

Alzheimer disease and related dementias can impact emotional distress and individual and relational health among persons living with dementia and their romantic partners. There are limited skill-based interventions that can help couples build resilience while navigating dementia-related challenges.

**Objective:**

We aim to assess the feasibility, acceptability, and preliminary signal of improvement of Resilient Together for Alzheimer’s Disease and Related Dementia (RT-ADRD), a novel dyadic skills-based intervention aimed at preventing chronic emotional distress early after diagnosis.

**Methods:**

Couples are randomized to complete either (1) six weekly conjoint intervention sessions of RT-ADRD or (2) a minimally enhanced usual care condition involving a self-guided pamphlet. Both partners must demonstrate capacity to consent to research participation, and at least 1 partner must exhibit clinically elevated emotional distress. Partners living with dementia must have received their diagnosis in the past 6 months. Participants complete baseline and posttest assessments of resiliency skills, emotional distress, relationship functioning, and quality of life and assessments of treatment satisfaction at posttest only.

**Results:**

This is aim 3 of a 5-year funded study that initially began in July 2022. Participant recruitment and data collection for this phase began in October 2024 and will continue until 40 couples (80 individuals) are randomized and complete the posttest. As of manuscript submission (July 2026), 25 couples have been enrolled and 25 couples have been randomized. Data collection is currently underway, and study completion is expected by early 2027.

**Conclusions:**

This study will gather comprehensive information on the feasibility and acceptability of RT-ADRD relative to minimally enhanced usual care and assess the preliminary signal of improvement in proximal outcomes. Findings will directly inform plans for a larger efficacy trial.

## Introduction

### Background

Alzheimer disease and related dementias (ADRDs) are significant and growing public health concerns with substantial personal costs for persons living with ADRDs and their romantic partners [1,2]. Individuals living with ADRDs experience early cognitive (eg, communication difficulties and memory changes) and behavioral disturbances (eg, mood and personality changes) that progressively compromise independence and daily functioning [3,4]. Although symptom trajectories vary, romantic partners (herein referred to as “care partners”) are frequently impacted by the onset of symptoms and resultant disruptions to routines and roles, thereby increasing stress for both partners [3], so much that they are often described as the “second patient” [5,6].

The period after receiving a formal ADRD diagnosis is one of increased risk for clinically elevated emotional distress (ie, depressive and anxiety symptoms) for individuals and their care partners [7]. Many (23%‐53%) individuals and care partners report elevated distress due to the overall poor prognosis of the condition, disruptions to their daily routines, and changes in roles and dynamics in their relationship. Over time, these challenges contribute to chronic distress and poorer well-being and prognosis [2,4,8,9] [10,11]. Recently, disease-modifying antiamyloid therapies have offered new avenues for postdiagnosis care in their ability to positively impact cognition [12,13]. However, these therapies currently can come with high out-of-pocket costs and can require frequent visits, limiting accessibility for many patients [14,15], and qualitative emerging research suggests that these novel care pathways can serve as a new source of stress without individualized support for treatment decisions [16].

Early intervention to address emotional distress in the period after an ADRD diagnosis is critical for several reasons. First, there is robust evidence that emotional distress is interdependent (ie, bidirectional and mutually transmitted) between partners [17]. Second, without intervention, distress can become chronic and more difficult to treat for individuals living with ADRDs and care partners [6]. Third, chronic emotional distress has a strong negative impact on each person’s individual and relational well-being over time, and distress can impair the quality of dyadic management of ADRD symptoms and disease progression [2,18]. Unfortunately, couples report unmet needs for programs that they can participate in together to adjust positively to the diagnosis—individually and as a unit [19-21].

Dyadic interventions can provide education and skill-based treatment to both members of a couple simultaneously, which may be a more acceptable, effective, and economical modality to build resiliency, reduce emotional distress, and preserve relationships and quality of life [22-24]. The few existing dyadic interventions for early-stage and moderate-stage ADRDs are generally focused on care partners planning and adjustment to symptoms, with evidence for reduced depressive symptoms and increased quality of life [25-27]. While beneficial, these programs do not provide interpersonal skill training to strengthen couples’ abilities to respond to daily dementia-related challenges as a team [28].

Dyadic interventions developed by our team show promise for reducing emotional distress among dyads navigating other neurological conditions early after diagnosis. For example, Recovering Together is a dyadic intervention aimed at preventing chronic emotional distress among patient-caregiver dyads recruited from neuroscience intensive care units following acute neurological injury [29,30]. We developed Recovering Together by leveraging prominent theoretical frameworks (ie, family strength vulnerability model, dyadic longitudinal model, and resiliency framework) and dyadic intervention methodologies (eg, longitudinal actor-partner interdependence modeling and dyadic clinical trial procedures) [29,31-34] and synthesizing available theoretical and empirical research to develop a transdiagnostic framework for adapting dyadic interventions to promote early dyadic adjustment to neurodegenerative diseases [23]. This provided a foundation to guide early iterations of a novel program in line with the National Institutes of Health (NIH) stage model for intervention development. Recovering Together has continued to demonstrate feasibility and preliminary efficacy, and it is currently being evaluated in a fully-powered efficacy trial [34]. Drawing on the success of Recovering Together, we identified specific adaptations that could address the unmet needs of couples early after ADRD diagnosis [35-37]. Our conceptual model, shown in Figure 1, showcases that the resiliency skills training embedded in this intervention is a dynamic process that targets both individual and interpersonal levels, fostering the ability to adapt effectively to significant adversity [38-40].

**Figure 1.**
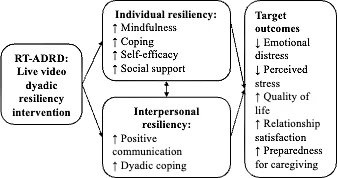
Conceptual model of the Resilient Together for Alzheimer’s Disease and Related Dementia (RT-ADRD) intervention for couples following Alzheimer disease and related dementia diagnosis.

Figure 2 describes the progress toward iterative development of Resilient Together for Alzheimer’s Disease and Related Dementia (RT-ADRD), a novel dyadic skill-based intervention aimed at preventing chronic emotional distress early after ADRD diagnosis. First, we conducted a qualitative study with dementia care specialist providers (n=19) to inform screening, referral, and recruitment procedures [41]. We also surveyed couples navigating a recent ADRD diagnosis (n=16 dyads) to understand preferences for dyadic programming (eg, format and content) and identify perceived barriers and facilitators to program engagement [42]. Feedback from these studies was used to develop the first version of RT-ADRD, which demonstrated feasibility and acceptability in an open pilot study (n=6 dyads) [43,44]. Exit interview feedback was used to further refine the intervention by making modifications to the program manual (eg, improved background sections of sessions and simplified worksheets) and restructuring the delivery of module content (eg, including website information with audio and video exercises alongside skills descriptions) to better align with participant needs and preferences.

**Figure 2.**
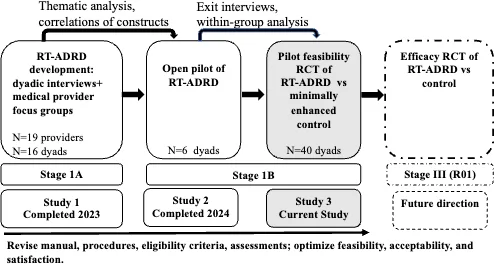
Iterative development of the Resilient Together for Alzheimer’s Disease and Related Dementia (RT-ADRD) intervention for couples following an Alzheimer disease and related dementia (ADRD) diagnosis. RCT: randomized controlled trial.

### The Present Study

This study protocol describes aim 3 of a comprehensive 5-year project aiming to adapt the existing research, methodology, program content, and procedures from the Recovering Together program to develop RT-ADRD. Our primary objective is to describe the protocol for the pilot feasibility randomized controlled trial (RCT; NIH stage model stage 1b) of RT-ADRD (Figure 2; study 3) to obtain additional information on the feasibility and acceptability of the intervention relative to a minimally enhanced usual care (MEUC) condition. We also sought to explore whether there is a preliminary signal of improvement in proximal intervention outcomes to inform sample size considerations and protocol refinements for a planned future efficacy trial.

## Methods

### Ethical Considerations

This study was approved by the Icahn School of Medicine at Mount Sinai Institutional Review Board (24‐00633; approved August 29, 2024) and is registered on ClinicalTrials.gov (NCT06619327). This study adhered to SPIRIT (Standard Protocol Items: Recommendations for Interventional Trials) guidelines (Checklist 1) for reporting of RCTs [45]. All participants provide electronic consent to participate before starting the program via the REDCap (Vanderbilt University) HIPAA (Health Insurance Portability and Accountability Act)–compliant web-based application using a hospital-approved e-consent feature. Prospective participants are given ample time to review the consent form and discuss the form with a trained research assistant, who provides detailed information about the study and answers any questions. Prospective participants will be reminded that they may decline participation and can withdraw at any time. Participants are given a copy of the consent form and invited to ask questions about their participation at any point over the course of the study. Participants are individually compensated with US $40 following completion of the pretest assessments and an additional US $40 after posttest completion, for a total of US $160 per couple per time point. Participants who partially complete the program will be paid for completing the pretest. All study procedures are conducted in accord with the ethical standards of the Committee on Human Experimentation of the institution in which the experiments are done or in accord with the Helsinki Declaration of 1975.

### Study Design

The primary objective of this pilot feasibility trial is to test the feasibility and acceptability of the RT-ADRD dyadic intervention versus MEUC. Participants will include couples comprised of (1) individuals with a recent ADRD diagnosis or a diagnosis of mild cognitive impairment (MCI) and/or major neurocognitive disorder suggestive of ADRD pathology; and (2) their romantic care partners. We will recruit 50 randomized couples (ie, 100 participants), with a target of 40 who complete pretest and posttest assessments. Data regarding feasibility, acceptability, and preliminary signal of improvement will be used to prepare for a larger efficacy trial.

### Inclusion and Exclusion Criteria

Eligible participants include couples who currently live together and are willing to be randomly assigned to participate in either the RT-ADRD or MEUC conditions. There are 5 additional eligibility criteria. Individuals living with ADRD must (1) have a recent (≤6 mo) diagnosis, be 65 years or older at the time of diagnosis, of either confirmed ADRD or MCI/major neurocognitive disorder suggestive of ADRD pathology as determined by medical chart review and provider referral; and (2) have cognitive assessment scores and symptoms via medical chart review consistent with early-stage ADRD, as determined by Clinical Dementia Rating [46] scale scores of 0.5 (“very mild”) or 1.0 (“mild dementia”) and Montreal Cognitive Assessment (MoCA) [47] scores of 17 to 25. If persons living with ADRD do not have cognitive assessment scores available, we will administer the MoCA to confirm eligibility. Individuals living with ADRD and their romantic partners must both (3) speak English; and (4) demonstrate understanding of the study and research protocol as determined by scores over 12 on the University of California Brief Assessment of Capacity to Consent (UBACC) [48]. At least one member of the couple must (5) demonstrate clinically elevated emotional distress as determined by scores of 7 or higher on either subscale of the Hospital Anxiety and Depression Scale (HADS) [49]. Couples will not be eligible if (1) either individual has a co-occurring terminal illness; or (2) the individual living with ADRD was diagnosed with forms of ADRD that would preclude participation (eg, highly symptomaticfrontotemporal Ddementia–behavioral variant) as determined by their referring providers.

### Study Procedures

Couples will be recruited from Mount Sinai Hospital clinics that provide clinical care for persons experiencing memory or cognitive changes associated with early ADRDs. The study team will circulate information about the study via flyers, presentations, and word-of-mouth referrals and will present the study to the medical staff at the clinics to explain the purpose of the study and potential benefits to participants, discussing best ways to facilitate referrals. The study team has additionally obtained a HIPAA waiver of authorization to prescreen medical records of patients treated in the dementia care clinical infrastructure to inform providers of potential eligibility after ADRD diagnosis disclosure visits. Providers will then share information about the study with couples, connect couples with the study team directly, and provide study materials (eg, flyers and contact information) for interested couples to contact the research team directly. Couples can also complete a brief survey via a secure REDCap platform (accessed via QR code or URL on distributed study flyers) to complete initial screening for eligibility before being contacted by the study team.

The study team will contact interested individuals via telephone to schedule a phone or live video call with a research assistant, who will describe the study and conduct eligibility screening. Research assistants will document when couples decline participation and reasons for refusal within a password-protected, deidentified dataset. If either couple member is deemed ineligible upon screening, the study team will document ineligibility in REDCap and provide the couple with a comprehensive list of local and national resources.

Upon confirming interest and eligibility, each couple member will review the consent form, which will include a description of all study procedures, information about potential risks and benefits of participation, and study contact information. The research assistant will remind individuals that they may refuse to answer any questions that make them uncomfortable and that they can discontinue their participation at any time. They will also be informed that if either person discontinues their participation, they will both be withdrawn from the study. Participants will be assured that study withdrawal will not compromise their medical care in any way. Participants will provide informed consent verbally and electronically via REDCap’s electronic signature feature. To ensure that both couple members have the capacity to consent, the research assistant will administer the UBACC (scores>12). Research assistants will receive extensive training and supervision to administer the UBACC from the principal investigator (SMB), a licensed clinical psychologist with experience working with individuals with cognitive impairment. Although participants are enrolled during the early stages of ADRDs, if study staff identify concerns regarding a participant’s decisional capacity during the course of the program, the principal investigator will evaluate the participant’s continued participation in accordance with IRB-approved procedures.

After providing informed consent, both couple members will be sent individual baseline questionnaires to complete separately via REDCap. The questionnaire will include measures assessing demographics, emotional distress, resiliency factors, and relationship functioning. Participants will also have the option of completing the questionnaires via Zoom with assistance from a trained research assistant using a screen sharing tool. Upon completing baseline questionnaires, couples will be randomized to RT-ADRD or MEUC via a 1:1 allocation ratio and a permuted-block–based sequence developed by the project statistical consultant (RAP) in REDCap. The randomization sequence is maintained within the REDCap production environment and is accessible only to the project statistician (RAP) and the designated data manager (Brittany Engelman) responsible for implementing the allocation. Study personnel responsible for participant screening, enrollment, baseline assessments, and outcome data collection remain blinded to treatment allocation. Block sizes are not disclosed to study personnel. Treatment assignment is revealed only after both members of the couple have completed their baseline assessments and randomization has been initiated within REDCap. If 1 member of the couple withdraws from the intervention, the dyad will discontinue the intervention due to RT-ADRD being delivered as a couple-based intervention. However, both participants will be given the opportunity to complete the postprogram assessments at the end of the 6-week period.

### Treatment Conditions

Couples randomized to RT-ADRD will attend 6 weekly treatment sessions led by an advanced clinical psychology doctoral student (ie, “program clinician”) with experience delivering behavioral interventions in medical settings. Program clinicians will be supervised by the principal investigator of the study, a PhD-level licensed clinical psychologist (SMB). Couples will receive both digital and physical copies of the program manual containing information and skills to review with the program clinician during their weekly RT-ADRD sessions. They will also receive a link to the RT-ADRD website containing video descriptions of skills and audio exercises to practice between sessions. RT-ADRD sessions will be audio-recorded to assess treatment fidelity.

Couples randomized to the MEUC condition will also receive a digital and physical copy of the self-guided pamphlet containing information about ADRDs, ways of navigating common challenges, and reflection questions to discuss together. They will receive weekly email reminders from the research assistant to review the materials together for 6 weeks. After completion of the treatment program, couples in both conditions will complete a posttest questionnaire containing the same measures as the baseline assessment as well as a measure of program satisfaction.

### RT-ADRD Intervention

#### Program Description

RT-ADRD includes six 30 to 60-minute sessions with a program clinician, which can be flexibly delivered in-person or via health care–system supported videoconferencing (per couples’ preferences) and scheduled in accordance with couples’ preferred dates and times. Couples will receive weekly email or text reminders (based on their preference) to prompt them to engage in the material together. Sessions can be rescheduled as needed. The RT-ADRD program website also contains the program manual, videos describing skills, and audio exercises to facilitate review and practice of skills outside of sessions.

RT-ADRD will provide individual and interpersonal resiliency skills training, with skills organized into 8 modules (Table 1). The skills—which are drawn from mindfulness, dialectical behavioral therapy, and cognitive behavioral therapy approaches—center on reducing interpersonal distress and enhancing self-efficacy, engagement of social support, and positive interpersonal communication [24,38,40,50,51]. In Sessions 1 to 3, all couples will complete RT-ADRD’s first 3 modules, each of which focuses on individual coping skills for common challenges early after diagnosis (eg, deep breathing, mindfulness meditation, and identifying dialectics). The skills in each session will be taught in a dyadic context and delivered uniformly to both partners. Program clinicians will work with couples to flexibly apply the content from each session to address the unique needs of each partner.

**Table 1.** Modules included in the Resilient Together for Alzheimer’s Disease and Related Dementia (RT-ADRD) intervention for couples affected by Alzheimer disease and related dementias in a pilot randomized controlled trial.

Modules	RT-ADRD topics	Content and skills^a^
1	Being in the Here and Now	Deep breathing, mindfulness, and present moment focus
2	Complex and Conflicting Experiences	Dialectics and mindfulness meditation
3	Managing Overwhelm	Distress spiral, open communication
4	Current and Future Challenges	Acceptance and change and cope ahead
5	Coping as a Team	Clarifying goals and we-based coping
6	Enhancing Support and Partnership	Navigating support and defining roles
7	Building Routines	Goal setting and valued activities
8	Acceptance and Meaning Making	Willingness and reflection

a All modules include home-practice of skills

Couples will collaborate with their program clinician to choose 3 of the 5 remaining modules, which focus on interpersonal coping and how couples can navigate ADRDs together as a unit (eg, clarifying goals, “we-based” coping), to comprise sessions 4 to 6. Importantly, all couples will have access to the program manual, videos, and audio exercises from all 8 modules via the program website, regardless of which modules are selected for sessions 4 to 6. This format allows the program to provide a core foundation of skills training and tailor its content to meet each couple’s specific needs and preferences.

#### MEUC Control

The MEUC condition is a 31-page booklet that includes educational information on topics including (1) accessing information, (2) difficult emotions, (3) changes in relationships, (4) ways of coping, (5) care partnership, and (6) maximizing support. The booklet also includes a list of local and national resources for the early period after ADRD diagnosis. Couples will receive both a physical and electronic version of the self-guided pamphlet, and opt-in to receive weekly email or text reminders to prompt them to engage in the material together.

### Treatment Fidelity

We will adhere to NIH recommended procedures to support fidelity [52]. The principal investigator (SMB) will lead weekly meetings to reinforce protocol adherence, address participant concerns, problem-solve issues, and prepare program clinicians for sessions. All sessions for the RT-ADRD condition will be audio-recorded, and a random sample (15%; 18 sessions total; 9 sessions per coder) will be coded by 2 independent coders (trained research assistants) to evaluate fidelity against pre-established checklists for each session developed by the research team.

### Assessments

Couples will independently complete a baseline and posttest survey via REDCap to assess intervention targets, hypothesized mechanisms of change, and potential moderators of intervention effects as illustrated in Table 2.

**Table 2.** Primary and secondary outcome measures and treatment targets assessed at baseline and postintervention.

Assessments, constructs, and descriptions	Baseline	Postintervention
Primary outcomes		
Feasibility		
Recruitment and consent: proportion of dyads who screen eligible that agree to participate in the intervention (benchmarks: acceptable>70%; excellent>80%)	✓	✓
Retention after randomization: proportion of dyads complete at least 1 session of RT-ADRD^a^ or 1 week of MEUC^b^ that complete posttest assessments (benchmarks: acceptable>70%; excellent>80%)		✓
Assessment: proportion of dyads without measures fully missing from baseline and posttest assessments (benchmarks: acceptable>70%; excellent>80%)		✓
Adherence to treatment: proportion of dyads who attend at least 1 RT-ADRD session attending 4 of 6 sessions (benchmarks: acceptable>70%; excellent>80%)		✓
Therapist fidelity: proportion of RT-ADRD sessions that are adherent to the program (benchmarks: acceptable>70%; excellent>90%)		✓
Acceptability		
Client Satisfaction Questionnaire (3 items): a measure of satisfaction with treatment program; evaluated in terms of scores over the midpoint (benchmarks: acceptable>70%; excellent>75%)		✓
Credibility and Expectancy Questionnaire (6 items): assess how credible dyads perceive the overall goal of programs in terms of scores over the midpoint (benchmarks: acceptable>70%; excellent>75%)	✓	
Demographics		
Medical chart review and participant self-report: age, sex/gender, race/ethnicity, education, and income	✓	
Clinical characteristics		
Medical chart review and participant self-report: type of ADRD^c^ diagnosis, time since diagnosis, and cognitive tests	✓	
Secondary outcomes		
Emotional distress		
Hospital Depression and Anxiety Scale (14 items): a measure of emotional distress (anxiety and depression subscales)	✓	✓
Geriatric Depression Scale (15 items): a measure of depression symptoms	✓	✓
Perceived stress		
Perceived Stress Scale (4 items): a measure of perceived stress	✓	✓
Quality of life		
WHO^d^ Quality of Life–Short Form (26 items): a measure of quality of life	✓	✓
Relationship satisfaction		
Couples Satisfaction Inventory (16 items): a measure of couple relationship satisfaction	✓	
Preparedness for caregiving (care partners only)		
Preparedness for Caregiving Scale (8 items): readiness for caregiving in relation to spouse	✓	✓
Intervention mechanisms		
Mindfulness		
Cognitive and Affective Mindfulness Scale (12 items): a measure of mindfulness skills	✓	✓
Individual coping		
Brief COPE^e^ (28 items): a measure of adaptive and maladaptive coping strategies	✓	✓
Self-efficacy		
General Self-Efficacy Scale (10 items): a measure of self-efficacy	✓	✓
Social support		
SSQR^f^, Short Form–Revised: a measure of satisfaction with social support (SSQR-N^g^, 6 items; SSQR-S^h^, 6 items)	✓	✓
Interpersonal communication and coping		
Dyadic Relationship Scale (10 items for patients, 11 items for caregivers): a measure of positive and negative dyadic interactions	✓	✓
Dyadic Coping Inventory (9 items): a measure of dyadic stress management	✓	✓

a RT-ADRD: Resilient Together for Alzheimer’s Disease and Related Dementia.

b MEUC: minimally enhanced usual care.

c ADRD: Alzheimer disease and related dementia.

d WHO: World Health Organization.

e COPE: Coping Orientation to Problems Experienced.

f SSQR: Social Support Questionnaire.

g SSQR-N: Social Support Questionnaire–Support Network Availability.

h SSQR-S: Social Support Questionnaire–Overall Satisfaction.

#### Primary Outcomes

The primary outcomes for this trial are feasibility, acceptability, credibility, and satisfaction. We will evaluate primary outcomes based on established benchmarks (Table 2) that must be met before continuing to a subsequent efficacy trial, consistent with the NIH stage model [35]. Benchmarks were established based on prior feasibility trials of behavioral interventions [29,33] (Table 2). Feasibility markers (recruitment, retention after randomization, assessment, adherence to treatment, and treatment fidelity) will be assessed at the couple level using proportions. Recruitment and consent are defined as the proportion of eligible couples who consent to participate (numerator=eligible couples who consent; denominator=all eligible couples that were approached). Retention after randomization for this trial is defined as the proportion of randomized couples who complete the assigned intervention period (ie, at least 1 RT-ADRD session or 1 wk of MEUC) and complete the posttest assessment (numerator=randomized couples completing the intervention/control period and posttest assessment; denominator=all randomized couples). Assessments is defined as the proportion of randomized couples with complete baseline and posttest outcome assessments (numerator=couples with no fully missing outcome measures at both time points; denominator=all randomized couples). Treatment adherence is the proportion of couples assigned to RT-ADRD who attend at least 4 of the 6 intervention sessions (numerator=couples attending≥4 sessions; denominator=couples attending at least 1 RT-ADRD session). Therapist fidelity will be assessed at the session level as the proportion of intervention sessions delivered according to the treatment manual (numerator=adherent sessions; denominator=all reviewed RT-ADRD sessions). Benchmarks for each feasibility outcome are provided in Table 2

In addition, we will use the proportion over the midpoint on established measures to assess treatment acceptability. Measures include the 6-item Credibility and Expectancy Questionnaire [53] at baseline, which is a measure of perceived credibility. We will also use the 3-item Client Satisfaction Questionnaire [54] at posttest as a measure of treatment satisfaction. We will also record the number of adverse events at least possibly related to the intervention, with “excellent” indicating no recorded adverse events and “acceptable” indicating minimal (<5) adverse events.

#### Secondary Outcomes

Given that RT-ADRD aims to address emotional distress and preserve couples’ relationships after ADRD diagnosis, we will also assess several secondary intervention targets at the individual level.

##### Emotional Distress

We will assess emotional distress using the 14-item HADS [49] and the 15-item Geriatric Depression Scale (GDS)–Short Form [55]. The HADS includes two 7-item subscales that assess depressive and anxiety symptoms, respectively. HADS items are summed into subscale and total scores (possible ranges: subscales 0‐21; total 0‐42). We will examine total and subscale scores separately, with subscale scores ≥7 indicating clinically elevated symptoms. The GDS, which is particularly suitable for our treatment population, assesses past week depressive symptoms using a binary rating scale (yes/no). GDS items are summed into a total score (possible range 0‐15), with scores ≥5 indicating clinically elevated symptoms.

##### Perceived Stress

The 4-item Perceived Stress Scale [56] will assess past month’s perceived stress. The items are summed (possible range 0‐16), with higher scores indicating greater perceived stress.

##### Quality of Life

The 26-item brief World Health Organization Quality of Life (WHOQOL-BREF) assessment [57] will measure perceived quality of life. The WHOQOL-BREF assesses quality of life across four subscales (physical health, psychological, social relationships, and environment). The first 2 items assess individuals’ overall perceptions of quality of life and health. WHOQOL-BREF items are summed and transformed into an interval of 0 to 100, with higher scores indicating greater perceived quality of life.

##### Relationship Satisfaction

The 16-item Couple Satisfaction Index [58] will assess relationship satisfaction. The items are summed (possible range 0‐81), with higher scores indicating greater relationship satisfaction and scores below 51.5 indicating notable dissatisfaction.

##### Caregiving Preparedness

The 8-item Preparedness for Caregiving Scale will be administered to care partners only. Scores on the items are averaged and range from 0 to 4, with higher scores reflecting greater caregiving preparedness.

### Intervention Mechanisms

#### Overview

We hypothesized that the following mechanisms would underline potential changes in target outcomes: mindfulness, individual coping, self-efficacy, social support, and interpersonal communication and coping as described within our conceptual model in Figure 1. The validated measures listed below will assess these hypothesized mechanisms.

#### Individual Resiliency

##### Mindfulness

The 12-item Cognitive and Affective Mindfulness Scale, Revised [59] will assess mindfulness. The items are summed (possible range 12‐48), with higher scores indicating greater engagement in mindfulness practices.

##### Coping

The 28-item Brief Coping Orientation to Problems Experienced [60], which contains 3 subscales (problem-focused, emotion-focused, and avoidant), will assess individual coping strategies. Scores for each subscale are calculated by averaging the scores of each item, with higher scores indicating greater use of that coping style.

##### Self-Efficacy

The 10-item General Self-Efficacy [(61] scale will assess perceived self-efficacy. The items are summed (possible range 10‐40), with higher scores indicating greater perceived self-efficacy.

### Social Support

The Social Support Questionnaire (SSQR) contains items assessing availability and satisfaction of support using 2-part questions, denoted as Support Network Availability (SSQR-N) and Overall Satisfaction (SSQR-S) [62]. The SSQR-N subscale (6-items) is measured by averaging all items assessing the number of people available to support participants in various aspects of their lives. The SSQR-S subscale (6-items) scores are calculated by averaging items assessing how satisfied participants are with the social support received, ranging from 1 to 6, with higher scores reflecting greater social support satisfaction.

### Interpersonal Resiliency

#### Positive Communication

We will use the 6-item positive dyadic interactions subscale of the Dyadic Relationship Scale [63] to assess positive communication in the dyadic relationship. Scores for the subscale range from 6 to 24 for patients and care partners.

#### Dyadic Coping

We will use the stress communication by self (4-items), common dyadic coping (5-items), and evaluation of dyadic coping (2-items) subscales from the Dyadic Coping Inventory [64]. Items are scored from 1 to 5 and summed to create subscale composites. Stress communication scores range from 4 to 20, common dyadic coping scores range from 5 to 25, and evaluation of dyadic coping ranges from 2 to 10, with higher scores on each subscale representing more positive evaluations of facets of dyadic coping.

### Covariates

We will include social determinants of health, using NIH common data elements, as covariates in our analyses of secondary outcomes and mechanisms. We will also explore associations with primary feasibility outcomes. These covariates will include socioeconomic status, education, social and medical support, age, race/ethnicity, biological sex, and gender [65].

### Data Analysis

This study was effectively unblinded for participants and study clinicians because the programs are not matched in terms of time or attention; however, the blinded statistician (TC) will conduct primary analyses using a dataset that has the conditions coded as “1” and “2”—which will be established by the unblinded statistician (RAP). Given the nature of the assessment, there will be no missing data for feasibility metrics for recruitment and consent, retention after randomization, assessment, adherence to treatment, or therapist fidelity. For missing data on self-reported acceptability outcomes (satisfaction and credibility), there will be no imputation, and acceptability will be determined based on the score without imputation. For exploratory treatment outcomes (emotional distress, perceived stress, quality of life, relationship satisfaction, and preparedness for caregiving) and mechanisms (mindfulness, individual coping, self-efficacy, social support, interpersonal communication, and interpersonal coping) with sparse missing data (less than 1 quarter of items), data will be imputed using mean imputation; otherwise, the scale will not be scored. If there is substantial missing data, or substantial differences in missing data by treatment group, we will test associations of key demographics and psychosocial variables with missingness and report any significant differences. We will report proportions for primary outcomes (feasibility, acceptability, credibility, and satisfaction) separately for RT-ADRD and MEUC (pooled for recruitment and consent). Feasibility markers will be compared to our prior dyadic intervention studies using proportions outlined in Table 2. Acceptability, credibility, and satisfaction will be evaluated with the proportion of individuals who score above the midpoint on self-report questionnaires.

This pilot RCT is not powered to evaluate signals of improvement in target outcomes and intervention mechanisms. In line with guidelines for feasibility studies, we will not assess the efficacy of the RT-ADRD condition relative to MEUC. However, we will explore preliminary signal of improvement and preliminary changes in hypothesized mechanisms and target outcomes using within-group 2-tailed *t* tests and Pearson correlations for the association of changes in mechanisms and outcomes. This will be conducted separately for individuals living with dementia and partners. We will examine effect sizes (Cohen *d*) with confidence intervals as a metric of the magnitude of observed effects. We will also qualitatively examine the direction and magnitude of differences between the RT-ADRD and MEUC conditions to determine whether there is evidence for a potential benefit of RT-ADRD compared to MEUC. The statistical mentor (RAP) will advise on the analyses and review the results.

### Trial Monitoring

Data monitoring will be handled by the principal investigator (SMB) and an independent safety officer approved by the funding agency (the National Institute on Aging [NIA]). The principal investigator and study team developed a detailed protocol for monitoring study procedures that was approved prior to the start of the awarded project; this protocol contains clear definitions for reporting of any adverse events (harms), protocol deviations, or changes to study procedures. Harms are defined consistently with the NIA Adverse Event and Serious Adverse Event guidelines [66]. If they occur, serious adverse events (adverse events that are life threatening or result in significant illness or injury) would be reported within 24 hours of the study team’s knowledge to the local IRB, the NIA-appointed safety officer, and the NIA-appointed program officer. If they occur, nonserious adverse events (ie, any untoward or unfavorable medical occurrence in the context of the study) or other unanticipated problems would be documented using the NIA Adverse Events form and shared with the safety officer annually. Given the minimal-risk nature of the study and primary focus on assessing feasibility and acceptability outcomes, there were no planned interim analyses or specified stopping rules prior to the start of the project. However, at any point during the study, the safety officer could determine (based on annual review and/or reported adverse events) that the participant risk outweighs participant benefit and choose to recommend study termination.

## Results

This study was supported by a grant from the NIA (1K23AG075188) to SMB beginning in September 2022. Participant recruitment and data collection for this phase began in October 2024 and will continue through mid-2026. Data collection is ongoing. As of manuscript submission (July 2026), we have recruited 25 dyads; 25 couples have been randomized; and we aim to recruit 50 dyads, with 40 completing pretest and posttest assessments. Anticipated completion of data collection and analysis is in late 2026. Study completion is expected by early 2027.

## Discussion

### Study Objectives

ADRDs are increasingly common and have devastating impacts on persons living with ADRD and their romantic care partners [2,18]. Currently, there are few resources that couples can participate in together early after diagnosis to reduce emotional distress and promote positive adjustment to an ADRD diagnosis [22,28]. To meet this unmet treatment need, our team used comprehensive qualitative feedback from providers, persons living with ADRD, and care partners to adapt an existing dyadic intervention and inform the development of RT-ADRD, a novel skills-based dyadic intervention for couples navigating a recent ADRD diagnosis [41,42]. Given that the adapted program demonstrated preliminary feasibility and acceptability in an open pilot [43], we now aim to evaluate the feasibility and acceptability of RT-ADRD as a necessary step prior to efficacy testing. If successful, the program will be the first dyadic resiliency intervention for couples at risk of chronic emotional distress after an ADRD diagnosis that includes information and skills training that partners can engage in together.

The present study describes the protocol for our pilot feasibility RCT trial of RT-ADRD relative to MEUC (an educational control condition) within NIH stage model stage 1b of intervention development. We will use these results to inform the subsequent stage—a fully powered efficacy trial of RT-ADRD relative to MEUC. Overall, this research will evaluate a novel intervention to address a critical gap in care that may be useful for preventing chronic emotional distress among couples navigating various disruptions early after an ADRD diagnosis. In the long-term, we hope to rigorously test, refine, and implement RT-ADRD in line with the NIH stage model [35].

### Limitations

We are currently only delivering RT-ADRD in English and with couples. This means that the program is not currently available to non–English speakers and other dyad types (eg, parents and children). These limitations will be addressed with parallel studies and in subsequent trials. Long-term, we hope to tailor RT-ADRD to meet the needs of linguistically diverse couples and other caregiving dyads. In addition, RT-ADRD is currently only delivered in a large academic medical center. Thus, RT-ADRD may need adaptations to promote successful implementation in other medical or community settings.

### Conclusions

The results from this pilot feasibility trial will be used to inform the efficacy testing of RT-ADRD, a dyadic resiliency program for those living with ADRDs and their romantic care partners, relative to an educational control condition. Our study will provide essential information surrounding the feasibility and acceptability of the program, as well as preliminary information regarding potential signals of improvement. These findings will be used to refine the procedures of the larger efficacy trial or to further refine and test the feasibility of the RT-ADRD program if the program does not meet established feasibility and acceptability benchmarks.

## Supplementary material

10.2196/106005 Checklist 1 SPIRIT checklist.

## Abbreviations

ADRD: Alzheimer disease and related dementia
GDS: Geriatric Depression Scale
HADS: Hospital Anxiety and Depression Scale
HIPAA: Health Insurance Portability and Accountability Act
MCI: mild cognitive impairment
MEUC: minimally enhanced usual care
MoCA: Montreal Cognitive Assessment
NIA: National Institute on Aging
NIH: National Institutes of Health
RCT: randomized controlled trial
RT-ADRD: Resilient Together for Alzheimer's Disease and Related Dementia
SPIRIT: Standard Protocol Items: Recommendations for Interventional Trials
SSQR: Social Support Questionnaire
SSQR-N: Social Support Questionnaire–Support Network Availability
SSQR-S: Social Support Questionnaire–Overall Satisfaction
UBACC: University of California Brief Assessment of Capacity to Consent
WHOQOL-BREF: World Health Organization Quality of Life
